# The power of unbiased phenotypic screens – cellulose as a first receptor for the Schitoviridae phage S6 of *Erwinia amylovora*

**DOI:** 10.1111/1462-2920.16010

**Published:** 2022-04-19

**Authors:** Ute Römling

**Affiliations:** ^1^ Department of Microbiology, Tumor and Cell Biology, Biomedicum Karolinska Institutet Stockholm Sweden

## Abstract

Bacteriophages, host‐dependent replicative non‐cellular entities which significantly shape the microbial genomes and consequently physiological and ecological properties of the microbial populations are exploited to restrict plant, animal and human pathogens. Unravelling of phage characteristics aids the understanding of the basic molecular mechanisms of phage infections which can subsequently lead to the development of rationalized strategies to combat microbial pathogens. In an unbiased screen to investigate the molecular basis of infectivity of the fire blight pathogen *Erwinia amylovora* by the lytic Schitoviridae phage S6, the biofilm extracellular matrix component cellulose has been identified as a cyclic di‐GMP dependent first receptor required for infection with the phage to possess beta‐1,4‐glucosidases to degrade the exopolysaccharide. This absolute receptor dependency allows maintenance of a phage‐microbe equilibrium with a low bacterial density.

Viruses are non‐cellular organisms that require a living host from all kingdoms of life for multiplication and persistence. Viruses share physicochemical features and regulation with membrane‐budding entities, exosomes in eukaryotes and outer membrane vesicles of Gram‐positive and Gram‐negative bacteria (van Dongen *et al*., 2016). With the number of viruses consistently exceeding by far the number of host cells, viruses and host cells coexist. Bacteriophages, viruses that infect bacteria, have shaped and shape bacterial genomes, host physiology and impact important biogeochemical and ecological functions such as significantly contributing to gene transfer and being major regulatory entities of nutrient cycles. Phages manifest their traits in the genomes and ancient phage‐derived components include plasmids and bacteriocins (Nakayama *et al*., 2000; Kamal *et al*., 2021). Lysogenic phages can alter biofilm formation and virulence by playing a role in attachment and immune evasion (Tinsley *et al*., 2006; Rice *et al*., 2009). A lysogenic phage has been shown to alter the human immune response upon vaccination (Manfredo Vieira *et al*., 2018). As a counterbalance to phage infection, a wide diversity of defence mechanisms, including physical barriers to reach the microbial surface, restriction–modification systems and CRISPR/Cas, are encoded by bacterial genomes in order to restrict phage infections (Arber and Linn, 1969; Barrangou *et al*., 2007; Doron *et al*., 2018; Cohen *et al*., 2019).

Phages and their components have been domesticated for a wide variety of genetic, biotechnological and clinical applications. Bacteriophages such as lambda, Mu, P1 and P22 have not only played determinative roles in the analysis of bacterial genomes they have also been the source of highly efficient genetic engineering tools for higher organisms (Kutter and Sulakvelidze, 2004; Yarmolinsky and Hoess, 2015). Phages, as single entities, as cocktails, genetically engineered or as purified components, also play a role as alternatives or supplements to antimicrobial treatment to restrict infection by human and plant pathogens since almost a century (Parfitt, 2005; Lin *et al*., 2017; Farooq *et al*., 2022). Established phages are characterized in more detail and novel phages with unique characteristics are urgently sought with large uncultivated phage populations as a pool (Dion *et al*., 2020; Kortright *et al*., 2020; Andrade‐Martinez *et al*., 2022; Busby *et al*., 2022).

The initial crucial step in phage infection is adherence to the microbial cell by phage tail spike or fiber proteins or alternatively dedicated receptors (Nobrega *et al*., 2018). Phages can thereby use a variety of protein or polysaccharide‐based host receptors such as the glucose moiety in teichoic acid of Gram‐positive and the terminal glucose in lipopolysaccharide of Gram‐negative bacteria (Bertozzi Silva *et al*., 2016), which contributes to the determination of host specificity and the host range.

*Erwinia amylovora* is a plant pathogen causing devastating fire blight disease in cultivated rosaceous plants worldwide. In an attempt to identify the receptor for the Schitoviridae phage S6, the unbiased transposon library screen identified the exopolysaccharide cellulose produced by *E*. *amylovora* CFBP 1430 as the first receptor required for successful infection. Subsequent analysis suggested that phage S6 encodes potentially two 1,4‐beta‐glucosidases to degrade the respective exopolysaccharide, with one of them experimentally verified. This initial study thus highlights the impact of assumption‐free experimentation and puts forward several relevant conclusions.

Cellulose is a seemingly simple macromolecule composed of the chemically most inert beta 1,4‐connected hexose glucose residues with cellobiose as the subunit. The resulting linear glucan chains produced in some bacteria by an array of cellulose synthases are arranged in parallel to form microfibrils. The complexity of cellulose is founded in the resulting highly physically and chemically inert crystalline microfibrils which are kept tightly associated by multiple hydrogen bonds. Side chains as phosphoethanolamine are often covalently attached to the glucose moiety (Spiers *et al*., 2003; Thongsomboon *et al*., 2018; Sun *et al*., 2018), which leads to an at least partially amorphous macromolecular structure (Zogaj *et al*., 2001; Nicolas *et al*., 2021). However, genes which regulate cellulose biosynthesis and can substitute the glucan chain such as the *bcsEFG* gene cluster of the class II cellulose biosynthesis operon of *Salmonella typhimurium* and *Escherichia coli* with BcsG as a lipid headgroup phosphotransferase are not encoded by the cellulose biosynthesis gene cluster and flanking genes in *E*. *amylovora* (Castiblanco and Sundin, 2018; Römling and Galperin, 2015). Among a variety of exopolysaccharides produced by plant‐associated bacteria, cellulose, with its production stimulated by plant products, has important ecological functions as it, for example, is mediating root attachment of symbionts and plant pathogens to promote infection (Robledo *et al*., 2012; Castiblanco and Sundin, 2018; Matthysse, 2018; Jones‐Burrage *et al*., 2019; Augimeri and Strap, 2015; Ausmees *et al*., 1999; Augimeri *et al*., 2015).

Bioinformatic analysis of the phage genome revealed that S6 codes for two 1,4‐beta‐glucosidases (gp094 and gp095) belonging to different glycoside hydrolase families (Henrissat *et al*., 1989). Subsequent biochemical analysis showed that the gp095, a two domain protein with a glycoside hydrolase family 5 domain at its C‐terminal part was experimentally verified to efficiently degrade the model substrate carboxycellulose (Knecht *et al*., 2022). Glycoside hydrolase family 5 proteins comprise a large diverse family including endo β‐1,4‐glucanases. However, the substrate for the cellulases, crystalline or amorphous cellulose, their mode of binding to the cellulose macromolecule and the detailed catalytic specificity as exo‐ or endoglucanases and the product outcome to be determined. Glycoside hydrolases family 48 members prefer amorphous and crystalline cellulose over the model substrate carboxymethylcellulose (Guimaraes *et al*., 2002). Those glycoside hydrolases might at the same time serve as receptors as demonstrated for the tail spike deacetylase of phage P22. Remarkably, the cellulose‐binding azo dye Congo red is an efficient inhibitor of phage infection, suggesting that the interaction with the cellulose glucan chain or fibrils either inhibits binding of the phage receptor or the catalytic activity of the cellulase or both.

Although cell surface‐expressed polysaccharides might be protective (Porter *et al*., 2017), phages of various families such as Myoviridae, Siphoviridae and exclusively Podoviridae are able to distinctively exploit capsular, biofilm extracellular and other polysaccharides as receptors (Pires *et al*., 2016; Canfield and Duerkop, 2020; Knecht *et al*., 2022). On the other hand, phages, as an integral part or attached to the phage particle, can associate a broad range of depolymerases able to degrade bacterial exopolysaccharides such as alginate, levan and amylovoran and the plant heteropolysaccaride pectin (Knecht *et al*., 2019), which can also provide the ground for infection with alternative phages in (Born *et al*., 2014). While those enzymes have been exploited for biotechnological applications, their intrinsic functions are often still unknown. Phages that degrade diverse forms of capsules, virulence factors of single bacterial cells which protect against the host immune system such as phagocytosis by macrophages and the attack of the complement system, have long been known, phages that address biofilm exopolysaccharides have been less characterized. The exopolysaccharide cellulose as an extracellular matrix component of *E*. *amylovora* biofilms is, among other exopolysaccharides such as amylovoran and levan, a virulence factor of *E*. *amylovora* (Castiblanco and Sundin, 2018). Although produced in planta, the discovery of the association of depolymerases with substrate specificity allows the identification of phages with similar receptor specificity or usage of other exopolysaccharides as receptors. Such are homologs of the gp095 cellulase also encoded by Podoviriae phages which infect various γ‐proteobacterial species such as *Pseudomonas fluorescens* and *Klebsiella pneumoniae* (Fig. 1).

**Fig. 1:**
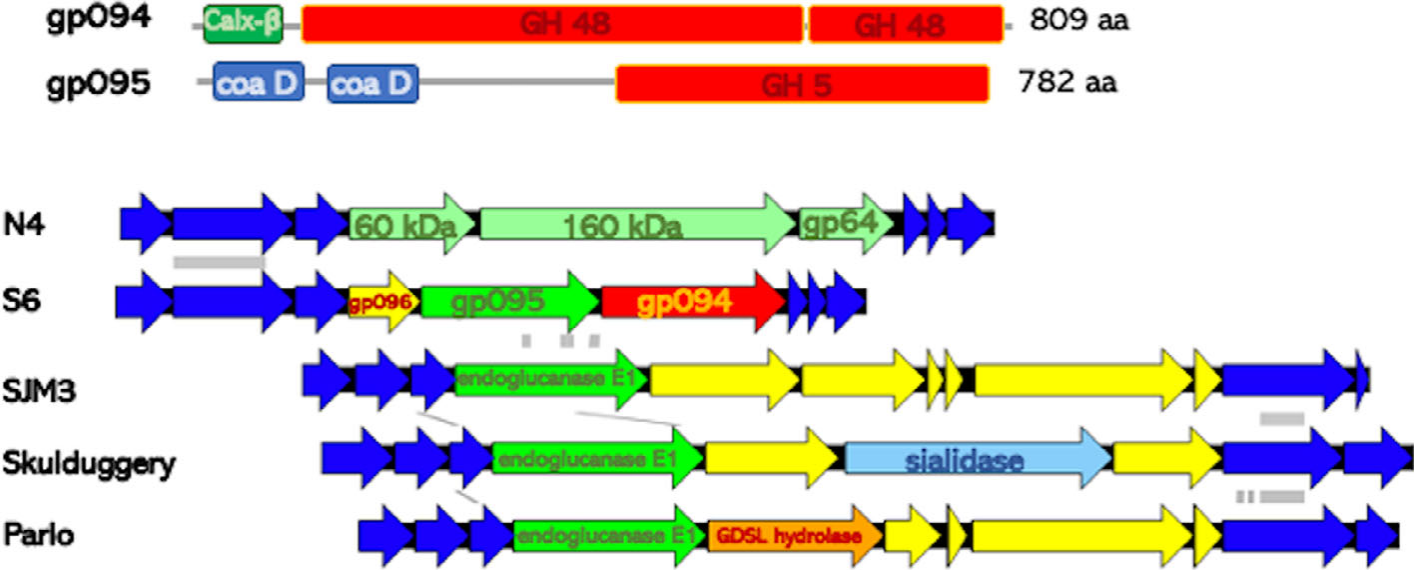
Domain structure and localization of glycosyl hydrolases of Schitoviridae phage S6. A. Domain structure of predicted gp094 and experimentally verified gp095 glucoside hydrolase. Gp094, annotated as a 1,4‐β‐cellobiosidase/endo‐processive cellulase, contains a N‐terminal Calx‐b domain functioning in Na+/Ca2+ exchange and a complete and partial glycoside hydrolase family 48 domain (Te'o *et al*., 1995). Predicted catalytic amino acids, the proton donor glutamate 155 and the catalytic base aspartate 331 are context dependent conserved. Gp095, annotated as an endoglucanase, contains N‐terminal two choice‐of‐anchor D domains of unknown function and a glycoside hydrolase family 5 domain. Predicted catalytic amino acids glutamate 570 and glutamate 689 are present. B. Localization of glycosyl hydrolases on the polymorphic region of S4 phage compared to the related N4 genome (Kiino and Rothman‐Denes, 1989; Sullivan *et al*, 2011). S4 encodes gp094 and gp095 glucoside hydrolases genes and gp096, a hypothetical protein in the polymorphic region. Homologs of the gp095 cellulase are encoded by the Podoviridae phage *Klebsiella pneumoniae* SMJ3, *Pseudomonas* phage Skulduggery and *Serratia* phage Parlo (Bockoven et al, 2019). Homologs of gp094 are encoded predominantly by *Streptomyces* and *Bacillus* sp (NCBI database accessed April 08, 2022). Genes of the polymorphic regions are colored alternatively to dark blue.

The identified β‐1,4‐glucosidases gp094 and gp095 are proteins with at least two domains. Besides the C‐terminal predicted exo‐ and endo‐glucosidase functionality respectively, the N‐terminal domains can be involved in the attachment of the proteins to the phage particle. It remains to be shown at which part of the phage particle these proteins are located.

Coincidentally, requirement of an exopolysaccharide for infectivity has recently been demonstrated not only for Schitoviridae phage S6, but also for the *Escherichia coli* N4 phage, an Enquatrovirinae family phage. Its host protein receptor NrfA, an outer membrane pore, has long been known to be required for infectivity and interaction with phage components (Kiino and Rothman‐Denes, 1989). More detailed recent studies unravelled that operon co‐encoded NrfB, a cytoplasmic membrane‐bound protein is a cellulose synthase‐like glycosyltransferase which, most likely, shares the substrate UDP‐N‐acetylmannosamine synthesized by the acetylglucoseamine 2‐epimerase WecB with the biosynthesis pathway for enterobacterial common antigen (Sellner *et al*., 2021). While the chemical identity and the ecological function of this exopolysaccharide macromolecule are still elusive, alanine substitution of catalytic residues of the processive glycosyltransferase showed that it is necessary for infectivity (Junkermeier and Hengge, 2021).

Interestingly, while a depolymerase has not been identified, the proposed tail spike deacetylase/mannase (Maffei *et al*., 2021; Sellner *et al*., 2021) interacting with the polysaccharide and NrfA is located in the corresponding polymorphic region of the N4 non‐segmented phage genome as the cellulases/glucosidases on the S6 phage genome. This observation suggests that in the case of N4, the exopolysaccharide is exploited as a first receptor, but is only modified and not degraded, while NrfA is the second receptor. It remains to be shown whether S6 possesses a second (protein) receptor in the outer membrane which, as the infectivity is stringently coupled with cellulose biosynthesis can be the translocation pore BcsC, which actually shows homology to NrfA and other outer membrane exopolysaccharide porins (Sellner *et al*., 2021).

The common denominator of the exopolysaccharide first receptors of S6 and N4 phage and prerequisite for infectivity is post‐translational stimulation of the catalytic activity of glycosyltransferases by the second messenger cyclic di‐GMP (Ross *et al*., 1987; Römling *et al*., 2000; Sellner *et al*., 2021). Cyclic di‐GMP is a ubiquitous second messenger in bacteria involved in motility to sessility lifestyle transition (Simm *et al*., 2004). While the cellulose synthase possesses a C‐terminal cyclic di‐GMP binding PilZ domain, NrfB has a C‐terminal MshEN domain, first recognized in AAA+ domain ATPases of type II secretion systems and pilus biogenesis nanomachines, which has been shown to bind cyclic di‐GMP with high affinity (Ryjenkov *et al*., 2006; Wang *et al*., 2016; Junkermeier and Hengge, 2021).

Distinct cyclic di‐GMP turnover proteins affect phage infectivity in a signal‐dependent way (Mutalik *et al*., 2020; Sellner *et al*., 2021). In the case of *E*. *amylovora*, the diguanylate cyclase HmsT, the homologue of which regulates the beta 1,6‐N‐acetyl‐d‐glucosamine exopolysaccharide in *Yersinia pestis* (Bobrov *et al*., 2008), has been identified to stimulate cellulose biosynthesis and to abolish phage infection upon deletion (Knecht *et al*., 2022). The EAL phosphodiesterase(s), which downregulate cyclic di‐GMP levels and cellulose biosynthesis in *E. amylovora* remain to be identified (Kharadi *et al*., 2018; Kharadi and Sundin, 2020).

On the other hand, N4 glucan biosynthesis and N4 phage infectivity are on the transcriptional and post‐translational level affected by multiple diguanylate cyclases and phosphodiesterases (Mutalik *et al*., 2020; Sellner *et al*., 2021). For example, the HTH‐EAL phosphodiesterase PdeL requires its catalytic activity to downregulate cyclic di‐GMP and its helix‐turn‐helix domain to repress transcription of the operon for the enterobacterial common antigen containing the substrate providing *wecB* gene (Sellner *et al*., 2021). Cumulatively, these results show that phage infectivity is highly dependent on environmental conditions and the growth stage of the microorganism.

With respect to applicability, the lytic phage S6 on its own does not display features that allow its control as a biocontrol agent for *E*. *amylovora* neither *in vitro* nor *in planta* as it does not eradicate the bacterial population but promotes coexistence with a low‐density cellulose deficient bacterial population. As cellulose is synthesized at high cell density the phage keeps a low bacterial population that might nevertheless be susceptible to infectivity by alternative phages where cellulose production otherwise would be a barrier. It remains to be demonstrated whether a phage cocktail including the S6 phage will effectively eradicate the pathogen *E*. *amylovora* without targeting ecologically important cellulose producing plant symbionts.
